# Modeling the Risk Factors of Undernutrition among Children below Five Years of Age in Uganda Using Generalized Structural Equation Models

**DOI:** 10.3390/children10121926

**Published:** 2023-12-14

**Authors:** Vallence Ngabo Maniragaba, Leonard K. Atuhaire, Pierre Claver Rutayisire

**Affiliations:** 1 African Center of Excellence in Data Science, College of Business and Economic, University of Rwanda, Kigali P.O. Box 3248, Rwanda; prutayisirec@ur.ac.rw; 2 School of Statistical Methods, College of Management Sciences, Makerere University, Kampala P.O. Box 7072, Uganda; latuhair@cobams.ac.ug

**Keywords:** undernutrition, under-fives, GSEM, paths analysis, Uganda

## Abstract

Introduction: The prevalence of undernutrition among children below five years of age, in Uganda and the world over, remains very high. About 45% of all global deaths among children below five years of age are attributed to undernutrition. A number of studies using different statistical approaches affirm this effect, yet some factors indicate the influence of other factors within the system. This study, therefore, uses a method that demonstrates how different variables feed into each other. Aim: The aim of this study was to establish the major factors associated with an increased likelihood of undernutrition and the paths showing how these risk factors influence undernutrition. Methods: Data from the Uganda Demographic and Health Survey (UDHS, 2016) were used for this study. A sample of 4530 children, whose age, height, and weight measurements were recorded, was considered for this study. Additionally, the study used generalized structural equation models to identify the multifaceted natures and paths of the risk factors that influence undernutrition among children below five years of age. The study relied on the UNICEF 2020 conceptual framework to identify and analyze the direct and indirect effects of these risk factors of undernutrition. Results: From the perspective of a male child, having a perceived small size at birth, a low birth weight, being breastfed for less than 6 months, having no formal education from mothers, limited income-generating opportunities, a low wealth status, and notable episodes of diarrhea were among the key factors associated with an increased likelihood of undernutrition. The identified paths were as follows: (i) Having no education, as this was associated with limited working opportunities and a low income, which increases the likelihood of low household wealth status, hence increasing the chances of undernutrition. (ii) Exposure to a rural setting was associated with an increased likelihood of undernutrition through association with poor and or low employment levels within the rural areas. (iii) A shorter duration of breastfeeding was associated with children in urban areas, resulting in an increased likelihood of undernutrition. (iv) Children aged between 6 and 47 months had a higher likelihood of undernutrition. Conclusions: An approach that addresses and recognizes all these factors at different levels, along the established paths, should be implemented to effectively reduce undernutrition among children below five years of age.

## 1. Introduction

Undernutrition results from inadequate intake of food nutrients relative to the body’s requirements. It can also result from an imbalance in diet or a failure of the body to absorb the required nutrients, leading to a poor linear growth rate of the child. A poor linear growth rate in children is manifested in three forms, namely, stunting (being too short for a given age), underweight (being too light for a given age), or body muscle wasting (being too light for a given height). A child experiencing at least one of these three outcomes (stunting, underweight, or body muscle wasting) is considered malnourished. Throughout the world, about 45.4 million children who are below 5 years of age experience underweight, 14.3 million experience wasting, while about 149.2 million (22%) experience stunting [1]. Undernutrition is worrying and should not be taken lightly, because about 45% of all deaths among children below 5 years of age are linked to it, with most of these deaths occurring within low- and middle-income countries, especially in Africa and Asia [2].

The dangers of undernutrition in children go well beyond physical impairments to slowing their cognitive and educational achievements [3]. For example, undernutrition in children in their early years is responsible for lowering their non-verbal IQ significantly [4,5]. Additionally, early childhood illness, like diarrhea, coupled with undernutrition, increases the likelihood of cognitive impairment in later childhood stages [6,7,8]. Undernutrition in childhood leads to physical weakness and poor performance at school [9] and at work in later stages of life, which leads to an increased likelihood of poor productivity at work, resulting in long-term consequences for the economy.

The prevalence of undernutrition varies across regions, societies, and across economic zones. For example, more than 90% of all stunted children are found only in Africa (41%) and Asia (53%); also, more than 95% of all children experiencing wasting are found only in Africa (27%) and Asia (70%) [1]. South Asia continues to be a global hub for child undernutrition, with a record high of over 35% of children stunted in the year 2017 [10]. Southeastern Asia follows suit, with over 27% and over 8% of all children stunted or wasted, respectively [11]. With an overall average of 13%, Latin America and the Caribbean are among the regions doing fairly well at managing undernutrition; however, the distribution across the region is unfair, as evidenced by the average rate of nearly 47% in Guatemala and over 20% in Haiti, Ecuador, and other Latin America and Caribbean countries [12]. In North Africa, more than 35% of children below five years of age experience undernutrition [13]; the Horn of Africa has about 7 million children below five years of age experiencing undernutrition and over 1.9 million children are at risk of dying from severe nutritional problems [13]. With more than 30% and more than 4% of all children below 5 years of age experiencing stunting and wasting, respectively, East Africa is among the regions in Africa and the world at large with a high prevalence of undernutrition [1].

Uganda, just like many other less-developed countries (LDCs), especially in Africa and Asia, has, time and again, experienced an unacceptably high rate of child undernutrition. At the national level, by 2016, the prevalence rates for the different categories of undernutrition among children below 5 years of age were as follows: 30 percent, or 2.4 million, stunted; 10 percent underweight; and about 4 percent of children wasted. With a prevalence of more than 31 percent [14], Uganda is among the countries in Africa, and the world at large, with the highest number of victimized children.

Undernutrition in Uganda, and in most parts of the world, has been analyzed without any attention paid to the nature of the variables that influence the problem. Little attention has been paid to the structural or causal mechanisms, pathways, or the structural system of causation [15,16,17,18,19], and this partly explains why undernutrition among children below 5 years of age, at 31.9 percent, is still unacceptably high and unpleasant. The structural mechanism of operation needs to be given much attention because undernutrition is multifaceted and complex in nature, and the factors influencing it operate at different levels in a hierarchical manner. Paying attention to the hierarchical nature of these factors, and to the fact that factors at lower levels feed into factors at the proceeding level, would help in more thoroughly understanding the complex problem of undernutrition, as well as its causes. Furthermore, the dynamics of the economy implore a continued examination of the prevalence of undernutrition and its risk factors using appropriate and advanced statistical methods such as GSEM and path analysis. Variables that influence undernutrition encompass, statistically, all nature of variables. While some variables were count, others were continuous or discrete, and the rest were categorically ordinal or nominal. Analytical methods that can accommodate all the variables as they naturally exist are prudent in the analysis of undernutrition. Such analytical approaches were missing in most earlier studies.

The current study recognized the hierarchical and the statistical nature of the risk factors of undernutrition among <5 s and is aimed at establishing the paths or the causal mechanism of the main determinants of undernutrition among children below 5 years of age in Uganda. Path analysis via generalized structural equation models (GSEMs) was an important scientific contribution to the world of literature on undernutrition and its causes. It reveals both the direct and indirect pathways through which different factors influence undernutrition among children below 5 years of age. Through path analysis, the most important risk factors for undernutrition among children below 5 years of age were identified and examined. And, the approach also helped in understanding the complex relationship between different risk factors of undernutrition. All in all, the study contributes to understanding the paths of the risk factors of undernutrition among children below 5 years of age in Uganda and informs the development of effective interventions to address undernutrition among children below five years of age in Uganda and beyond.

## 2. Methods and Materials

### 2.1. The Study Area and Data

**Figure 1:**
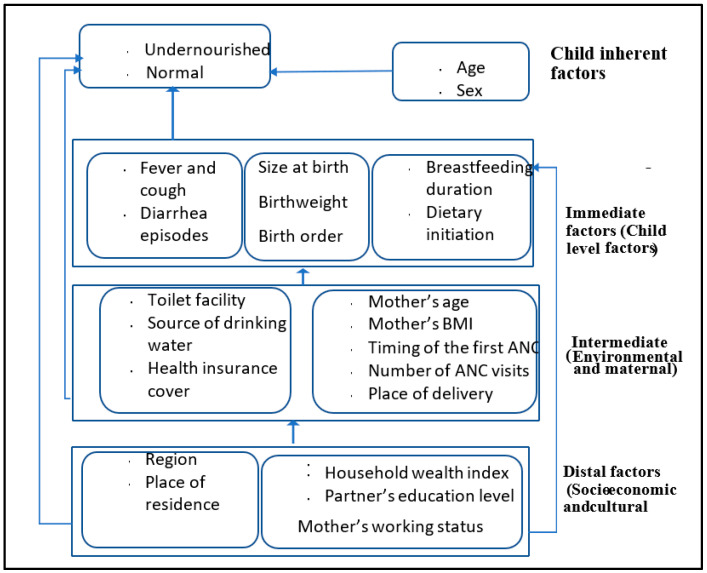
A conceptual framework based on the UNICEF Conceptual Framework on the Determinants of Maternal and Child Nutrition, 2020.

The study was conducted in Uganda, a country in East Africa. The study was based purely on secondary data which were mined from the Uganda Demographic and Health Survey (UDHS) for the year 2016. Data for children below five years of age in Uganda were extracted from the Kids Record (KR) file, one of the files within the UDHS database that contains data of children aged 0–59 months. The UDHS, 2016 covered the entire country and was administered within the 15 cluster regions, namely the South Central, North Central, Busoga, Kampala, Lango, Acholi, Tooro, Bunyoro, Bukedi, Bugisu, Karamoja, Teso, Kigezi, Ankole, and West Nile regions. The sampling frame used in the UDHS, 2016 was the sampling frame of the National Population and Housing Census (NPHC, 2014) [20]. The UDHS, 2016 employed a two stage cluster-sampling technique, with the primary sampling units being the enumeration areas (EAs), while the secondary sampling unit was the household [15]. The survey was keenly carried out by a team of experts who collected and processed data for 29,226 children and successfully took and recorded anthropometric measurements for about 4530 children below five years of age. Data were collected on several other factors, namely household socioeconomic factors, environmental factors, maternal related factors, as well as community level factors as displayed in (Figure 1).

The main objective of the study was to identify the most statistically significant risk factors of undernutrition and the paths through which they operate to increase the likelihood of undernutrition among children below 5 years of age in Uganda. In accordance with the UNICEF conceptual framework (Figure 1) for determinants of undernutrition among <5 s, a GSEM approach was proposed and consequently estimated using GSEM approaches.

The overall dependent variable was undernutrition of the child, which was proxied by the Composite Index of Anthropometric Failure (CIAF) that was derived based on various studies [21,22,23] in the literature. The World Health Organization observes stunting, underweight, and wasting as the three forms of undernutrition and, accordingly, any child with at least one of the three forms of undernutrition was considered undernourished. It was therefore prudent to construct the CIAF, a representative numerical value of the three components (stunted, underweight, and wasted), to represent undernutrition. Subsequently, as was proposed and used by earlier scholars such as Svedberg [24], the CIAF was derived based on the z-scores (Zis) of stunting, underweight, and wasting. All children whose z-scores fell below -2SD from the median of the reference population were categorized as undernourished (coded as 1), while all children whose z-scores were equal or above -2SD from the median of the reference population were categorized as normal (coded as 0). Henceforth, the CIAF was derived from all three forms of undernutrition by summing up the codes and, therefore, a sum of at least 1 indicated that the child had at least one anthropometric failure, and hence was undernourished, while a sum of 0 indicated a normal child with none of the three anthropometric failures.

As displayed in Figure 1, and based on the UNICEF, 2020 conceptual framework on the determinants of maternal and child nutrition [25], the risk factors of undernutrition operate at four levels, namely, the distal factors, the intermediate factors, the immediate factors, and the child inherent factors. The risk factors feed into each other(s) at different levels from the distal through intermediate, the immediate, or child level factors to influence undernutrition. The child inherent factors are unique in this system; they are not influenced by any other factors and do not influence any other factor but undernutrition in the system. Accordingly, structural equation models (SEMs) in the generalized form (GSEM) were found the most appropriate methods to analyze the problem of undernutrition among children below 5 years of age.

### 2.2. Model Building and Estimation Methods

In accordance with Figure 1, the interrelationship between different variables and their mode of influence on undernutrition needed to be observed keenly and were thus analyzed with a model that treats every variable as it is within the system. We observed that the variables operate at different levels and influence undernutrition either directly or indirectly. We also observe that some variables are influenced by others within the system. The Structural Equation Model (SEM) was the system of modeling that was found to be the most appropriate in establishing both the direct and indirect effects of explanatory variables on undernutrition. As observed from the conceptual framework, the key feature of the UNICEF, 2020 conceptual framework emphasizes an analysis that recognizes the fact that variables feed into each other at different levels and that their effect on undernutrition is either direct or indirect. Since we have a mix of all statistical variables, then a model that can accommodate all of them in a single analysis is prudent. GSEM has the power to analyze a mix of count, continuous, and categorical data while accommodating their hierarchical nature as well [21] and was thus found the most appropriate; hence, it was used for this study.

**Figure 2:**
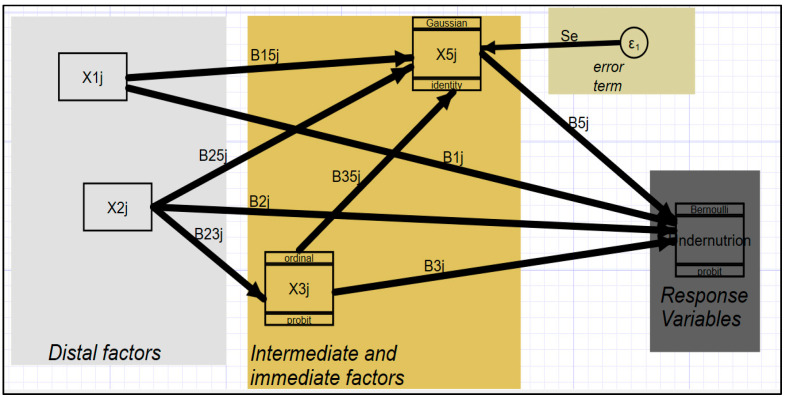
Illustration of GSEM analysis approach.

More so, the hierarchical and interrelated nature of the undernutrition risk factors calls for a model that incorporates and can estimate a system of equations simultaneously. Within the GSEM system, several nodes contain response variables and, thus, apart from the overall dependent variable of undernutrition, there were other response variables which were to be simultaneously estimated as illustrated in Figure 2.

The illustration in Figure 2 displays a structural or paths model that demonstrates, in a basic way, how several risk factors interact to influence each other as well as influence the response variable(s). Xij is the vector of variables at a given hierarchical level and, consequently, Bij is the vector of coefficients showing the association between variable i and j. The arrows represent the direction of effect from an explanatory variable to the response variable. The methodology requires a clarification of the nature of a variable, be it count, discrete, continuous, ordinal, or nominal. Accordingly, for GSEM, the process required a declaration of both the family (e.g., ordinal, Bernoulli, Binomial, etc.) and the link functions (e.g., Probit, identity, etc.) depending on the nature or statistical nature of the generalized response variable in the model. Per se, according to Figure 2, undernutrition being a binomial variable of Bernoulli type, with 0 for “no” anthropometric failure (normal child) and 1 indicating “yes” or a child with at least one anthropometric failure, dictates family (Bernoulli) with link function (Probit). All quantitative variables, if any, in the model belong to Gaussian family with Identity as the link function. The error term (ὲ) is generated every time the response variable is quantitative in nature with Gaussian and identity as the family and link functions, respectively.

GSEM is a family of statistical techniques that is powerful in the analysis of multivariate, multi-level, nominal, count, continuous, categorical, and ordinal data. It allows the analysis of a wide range of variable responses in the same model. It is very vital in the analysis of complex linkages of variables in models where variables are either exogenous, endogenous, or both. The effects of variables in the complex structure of the model are measured or determined simultaneously. Generalization was important in understanding the structural and simultaneous nature of the interaction of variables in undernutrition problems. GSEMs represent a generalization of SEMs and have the advantage of accommodating all types of variables by considering their link and family distributions [26]. GSEM also has other advantages over other modeling approaches, such as factor analysis (FA), since it relaxes the assumption of normality of variables and it allows for multi-level or hierarchical modeling as well as group analysis.

More so, GSEMs combine the power and flexibility of both structural equation models (SEMs) and generalized linear models (GLMs) in a unified modeling framework [27] and are a combination of the statistical distribution of the family (*F*) and the link function g (·) for the response variables, as clearly specified within the structural framework.

Thus far, in the current study the linear model of the format *yi* = *β*′*xi* + *ui*, where *x* is a vector of exogenous variables, was generalized to the linear model of the form *g*{*E*(*yi*)} = *β*′*xi* with *yi*~*F*. The idea of family and link functions was specified for endogenous variables of every equation in the system of the model (Figure 2). Sampling weight was used to account for disproportionality in the samples caused by survey design and, accordingly, the women’s individual sampling weight (v005) in the UDHS dataset was used as a weighting factor during the GSEM process. Throughout the study, the analysis was conducted using SEM building found within STATA version 15 while paying attention to the generalized nature of variables. Outputs of the analysis were in the form of both tables (showing both direct and indirect effects) and figures that were displaying the path and coefficients of explanatory variables on response variables in the system.

## 3. Results

**Figure 3:**
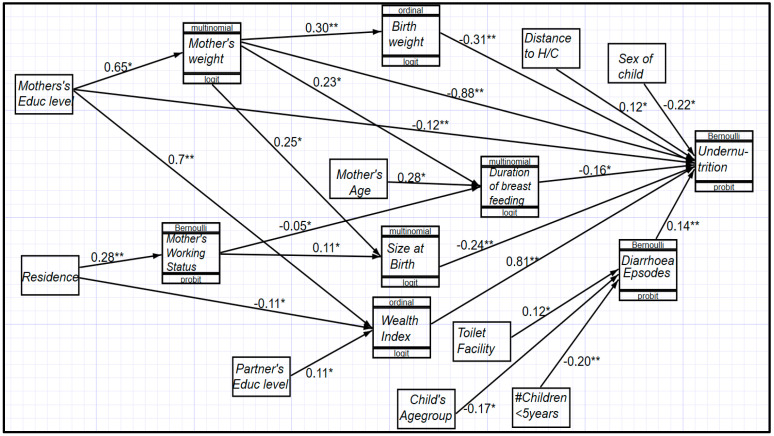
The generalized structural equation model showing the causal mechanism. (* = significant at 10%, ** = significant at 5%).

The interrelationship between undernutrition and its risk factors as depicted by the conceptual framework (Figure 1) and illustrated in Figure 2 implied an estimation of both the direct and indirect determinants of undernutrition with an aim of establishing the path and causal mechanism of the risk factors. The estimated results were presented in both the tables (Table 1 and Table 2) and the path diagram (Figure 3). Variables were judged at a 5 percent level of significance (**), though in the path diagram even variables which were statistically significant at 10 percent (*) were maintained in the figure. As such, a variable was considered to be a significant influencer of undernutrition whenever its *p*-value (p(z)) was less than or equal to 10% (0.1).

A total of 4530 cases were entered into the GSEM, but due to incomplete information for different variables on some of the cases, only 1731 cases had full information and were thus successfully considered in the final results. For the GSEM, any case found with missing information for any variable information was deleted. From Table 1, the sex of the child was statistically significant in influencing undernutrition among children below five years of age in Uganda. At the immediate level, perceived size at birth, birth weight, and duration of breastfeeding were found to be statistically significant; at the intermediate level, the mother’s weight as indicated by BMI was statistically significant, while the distal or socioeconomic factors that significantly influenced undernutrition among children below 5 years of age were the mother’s education level, distance to a health center, and the household wealth index. It was necessary to disaggregate the analysis via the variable categories in order to establish how categories within different risk factors influenced undernutrition. Accordingly, the results for the detailed categorical effects are indicated in Table 2.

Table 2 displays variable categories that were significantly associated with undernutrition. For all observations included in this table, the presented categories were significantly different from the base categories shown with asterisks (*). Respectively, the reference categories were age group (0–5), low birth weight, first wealth quintile, low BMI for mothers, breastfeeding for less than 6 months, small birth size, no education level, short distance to the nearest health center, and the male sex of the child. In this table, only results for those categories that were statistically significant from the base categories were presented. As observed from Table 2, the sex of the child, the mother’s education level, the duration of breast feeding, the mother’s body mass index, the perceived size at birth, the distance to a health center, the wealth index of the household, the weight at birth, and the age group of the child were significantly associated with undernutrition. Based on specific reference categories, which were normally the first category of each variable of concern, the following observations were made. The association between undernutrition and sex of the children was significantly different between male and female children. Being a male child was associated with an increased likelihood of undernutrition, while being a female was associated with decreased chances of undernutrition. The association between the mother’s education level and undernutrition in children was significantly different between mothers with no education and mothers with a primary level of education and above. Specifically, having no formal education was associated with increased chances of undernutrition. Likewise, a perceived low size at birth, having no formal education, being in the first and second wealth quintile, and a low birth weight were associated with an increased likelihood of undernutrition. Age groups of 6 months and above, breastfeeding beyond two years, and distance to a health center being a big problem were also associated with an increased likelihood of undernutrition.

Further analysis commenced with the aim of establishing the paths of the risk factors that were significantly associated with undernutrition among children below five years of age in Uganda. The path diagram for the statistically significant risk factors of under-fives’ undernutrition was estimated with the GSEM building approach with STATA 15.0, as displayed below (Figure 3).

Observations made from Figure 3 indicate that, among others, the factors that play a role in the path from risk factors to undernutrition include the age group of children, the number of children below five years of age in a household, partners’ education level, residence, mothers’ education level, mothers’ age, size and weight at birth, the duration of breastfeeding, diarrhea episodes, mothers’ BMI, the birthweight category of the child, the household wealth index, the availability of toilet facilities, the distance to a health center, and the sex of the child.

Less breastfeeding, a mother of a young age, low income-generating opportunities, a low BMI in the mother, rural settings, and mothers having no formal education level were associated with an increased likelihood of undernutrition. More so, poor household wealth was associated with an increased likelihood of undernutrition. At the same time, the factors that increased the likelihood of poor household wealth included a rural setting of the household and low education levels of both the mother of the child and her partner. A low weight of the child at birth was associated with an increased likelihood of child undernutrition. In the same line, low education levels of the mother were associated with poor household wealth, and hence an increased likelihood of undernutrition.

Briefly, some of the possible identified causal mechanism of undernutrition may operate through the following:

(i) Having no education was associated with limited working opportunities to generate some income, which increased the likelihood of a low household wealth status and, hence, increased the chances of undernutrition.
(ii) Rurality was also associated with an increased likelihood of undernutrition through association with poor and/or low employment opportunities within rural areas.
(iii) A shorter duration of breastfeeding was a common phenomenon in urban areas and was associated with an increased likelihood of undernutrition in such areas.
(iv) The rest of the factors, sex of the child and distance to a health center, are purely exogenous in this model.
(v) Children who were aged 6–47 months had an increased likelihood of undernutrition, especially those who were in unhealthy environments, such as poor households or environments with no toilet facilities, since these environments were associated with an increased likelihood of diarrhea episodes. The age category from 48 to 59 months turned out to be a statistically insignificant factor in influencing undernutrition among children below 5 years of age in Uganda.

## 4. Discussion of the Results

In this section, the results from this study are discussed in line with the literature on the subject matter. The analysis comprehensively depended on GSEMs and the conceptual framework for determinants of undernutrition among <5 s. The most statistically significant risk factors included the sex of the child, perceived size at birth, birth weight, duration of breastfeeding, distance to a health center, mothers’ weight, mothers’ level of education, and the household wealth index, and these are discussed as indicated below.

Deeper analysis of the sex of the child indicated that female sex, as opposed to male sex, was negatively associated with undernutrition among <5 s in Uganda. Earlier studies as well indicate that the probability of being undernourished was found to be significantly lower for female as compared with male children [26,27,28,29]. The duration of breastfeeding was a statistically significant risk factor of undernutrition in Uganda. Breast feeding is key in building the body’s immunity against childhood-related diseases and, as such, the more the child breastfeeds, other factors constant, the more immune they are. Exclusive breastfeeding is necessary in the first 6 months after birth as it is influenced by better growth but care must be observed when introducing complementary feeding [30]. The age of weaning is key in reducing undernutrition [30], while undernutrition increases with an increase in the age of the child [31]. In line with the current findings, the size and weight of the child at birth were statistically significant in influencing undernutrition, and this is in agreement with the findings of many other scholars [32,33]. The mother’s weight (BMI) and age at first birth increased the likelihood of a child’s undernutrition in the current study and was similar to the findings of other studies from other countries [34,35,36]. Undernutrition may be negatively associated with the mother’s weight; for example, undernourished children were found within households with obese mothers [37,38]. While there is no direct evidence that maternal obesity causes child undernutrition, there is evidence that maternal obesity is associated with a higher risk of adverse birth outcomes, such as preterm birth and low birth weight [39], and, most often, these outcomes lead to child undernutrition.

The number of children in the household correlates to the level of dependents and, thus, the pressure to facilitate and provide care and food. The portrayed results reveal that the number of children below five years of age within households strongly and positively influences undernutrition and is also confirmed by some other scholars [40]. The rank of a child’s birth is associated with undernutrition, as the current findings affirm that the third-born children are the main victims of undernutrition in a household. Several studies have found an association between the rank of a child’s birth and undernutrition; for example, a population-based cross-sectional study in Shanghai, China found that a child’s odds of undernutrition were higher as the birth order or number of siblings increased [41], and there was also a similar case with a study in Mumbai and Bhubaneswar, India [42].

The education levels of parents are important in influencing childcare practices. The current study reveals a positive association between mothers’ formal education level and the undernutrition status of their children. The same association was also observed when the partners’ formal level of education was factored into the model. Improving mothers’ education levels as well as the education levels of their partners would help in reducing undernutrition among children below five years of age [40]. In most cases, the education of girls and mothers improves their employability skills and increases the odds of finding employment, earning money, and being self-reliant in upkeep and childcare practices. However, in some countries, mothers’ employment tends to increase undernutrition in under-fives [43,44]. The working status of mothers negatively influenced undernutrition of their children, implying that the more chances mothers had to work, the lower the rates of undernutrition [45]; however, according to many other scholars, mothers’ working and employment conditions implied diversion of time from children to work such that so that time to care for young children is reduced hence increasing the likelihood of being undernourished [46].

Household wealth and poverty are key determinants of households’ wellbeing. The wealth index correlates well with the level of household provisions and significantly influences undernutrition, not only in Uganda but globally as well [47]. Residence also influences undernutrition because urbanization influences the provision of many other social amenities that influence child wellbeing. As such, children in rural areas are at a higher risk of undernutrition than children in urban areas [47,48,49].

The paths through which factors influence each other at different levels is key in understanding the undernutrition problem and its risk factors. Improving the socioeconomic status, like education, influences employment opportunities for households and, hence, household wealth [50]. Improved education also influences the health-seeking behavior of women [51] and the potential to utilize health care services [43,51] which, in turn, influences the healthy living of mothers, especially the expectant ones. Health living of expectant mothers influences the optimal weight and size of the baby at birth [52], which is important in reducing undernutrition among under-fives [53].

From the model point of view, the current study was able to establish the paths and interlinkages between variables at different hierarchical levels using the GSEM. GSEMs have been used in several studies to analyze undernutrition with success in establishing direct and indirect effects. For example, studies conducted in Ethiopia and India [54] used GSEM and path analysis to identify the correlates and determinants of child undernutrition and morbidity, in which case birth size mediated the association between short-birth interval and undernutrition [55]. In Tanzania, a GSEM was used to investigate the factors associated with undernutrition, and it was revealed that women’s employment had the potential to improve their nutritional status [56].

## 5. Conclusions

Undernutrition among children below five years of age in Uganda is still a major public health problem that needs to be handled with much effort. This study reveals that the factors that influence undernutrition are multi-faceted and complex, and they feed into each other at different levels, ranging from distal, intermediate, immediate, child level, and child inherent factors and, as such, nutritional interventions should take into consideration the paths and the interaction mechanism of the risk factors. The strategies and policies against undernutrition should consider the interconnectedness of the risk factors and, hence, simultaneously work on all the factors along the identified path(s).

## 6. Strength of This Study

One of the strengths of this study lies in its ability to perform causal inference. The use of path analysis via generalized structural equation models (GSEMs) allowed researchers to establish causal relationships between risk factors and undernutrition among children below 5 years of age in Uganda. Understanding the paths of the most statistically significant risk factors was important in designing effective interventions to address undernutrition both in Uganda and the world over. Another strength of this study lies in its comprehensive analysis. The use of path analysis via generalized structural equation models (GSEMs) allowed the researchers to analyze multiple risk factors simultaneously and assess their relative importance in influencing undernutrition among children below 5 years of age in Uganda. This approach can be extended to study several other epidemiological phenomena.

## 7. Limitations of This Study

One of the limitations of this study was the lack of the most recent dataset: The most recent and available data that this study could use was the UDHS, 2016. As such, the results reflect more of the situation of undernutrition in the year 2016. However, these results were important in t way that they provided key facts that making policies and making future projections can be based on. Another limitation to this study was the recall bias. Apart from the anthropometric measures which were measured, several other variables in the UDHS dataset were self-reported and, as such, the data may have been candidates for recall bias. Having a large enough sample size was one way that we aimed to reduce this limitation and improve the accuracy of the results. Finally, there was a sampling bias in the survey because it was not complete enumeration. Although the UDHS is a nationally representative survey that covers most parts of the country, there may still be some sampling bias in terms of the inclusion and exclusion criteria. This may have led to either over-representation or under-representation of some individuals. Using sampling weights was one of the methods we used to minimize sampling bias in this study.

## Figures and Tables

**Table 1:** GSEM-based determinants of undernutrition among children <5 years in Uganda.

Variable and Category Level	Coefficient	P >|z|	[95%Conf. Interval]
**Child inherent level**			
Sex	−0.333	0.000	−0.494 −0.171
**Immediate or child level factors**			
Birth weight	−0.296	0.003	−0.489 −0.103
Birth size	−0.308	0.004	−0.516 −0.100
Breast feeding duration	0.183	0.001	0.078 0.287
**Intermediate (environmental and maternal) level factors**			
Mother’s BMI	−0.020	0.000	−0.067 −0.772
**Distal factors**			
Wealth index	−0.126	0.001	−0.198 −0.054
Mother’s educ	−0.125	0.076	−0.263 0.013
Distance to HC	0.196	0.021	0.029 0.362

**Table 2:** Disaggregated categories of the risk factors of undernutrition among <5 s in Uganda.

Risk Factors of Undernutrition	Coefficient	z	P > |z|
**Age group**			
0–5 *			
6–11	0.365	2.13	0.033
12–23	1.000	4.22	0.000
24–35	0.729	2.66	0.008
36–47	0.741	2.62	0.009
**Birth weight category**			
small *			
normal	−0.344	−2.72	0.007
big	0.556	2.88	0.004
**Wealth index**			
first *			
middle	−0.249	−1.71	0.088
fourth	−0.466	−2.93	0.003
highest	−0.900	4.43	0.000
**Duration of breast feeding**			
0–11 *			
>24 months	0.940	2.80	0.005
**Perceived size at birth**			
small *			
large	−0.342	−3.08	0.002
**Mother’s education level**			
no education *			
primary	−0.372	−2.44	0.015
higher	−0.495	−1.68	0.093
**Distance to a health center**			
not a big problem *			
big problem	0.204	2.25	0.025
**Sex of the child**			
male *			
female	−0.325	−3.76	0.0

* (Base category).

## Data Availability

The data used for this study are available on request from the DHS program database at “The DHS Program—Uganda: Standard DHS, 2016 Dataset”, accessed on 14 October 2021.
